# Heteroatom-Doped Hierarchically Porous Biochar for Supercapacitor Application and Phenol Pollutant Remediation

**DOI:** 10.3390/nano12152586

**Published:** 2022-07-28

**Authors:** Diyong Tang, Li Lu, Zhipeng Luo, Baokun Yang, Jun Ke, Weidong Lei, Hongran Zhen, Yuan Zhuang, Jie Sun, Ke Chen, Jie Sun

**Affiliations:** 1 Key Laboratory of Resources Conversion and Pollution Control of the State Ethnic Affairs Commission, College of Resources and Environmental Science, South-Central Minzu University, Wuhan 430074, China; 2021110590@mail.scuec.edu.cn (L.L.); 2020120750@mail.scuec.edu.cn (Z.L.); 2020110555@mail.scuec.edu.cn (B.Y.); 2021120875@mail.scuec.edu.cn (W.L.); 202021241174@mail.scuec.edu.cn (H.Z.); 3047207@mail.scuec.edu.cn (J.S.); kechen@mail.scuec.edu.cn (K.C.); jetsun@mail.scuec.edu.cn (J.S.); 2 School of Chemistry and Environmental Engineering, Wuhan Institute of Technology, Wuhan 430073, China; jke@wit.edu.cn; 3 Experimental Teaching and Engineering Training Center, South-Central Minzu University, Wuhan 430074, China; 8800319@mail.scuec.edu.cn

**Keywords:** biochar, supercapacitor, organic pollutant, peroxydisulfate, free radicals, non-radical pathway

## Abstract

Biochars are considered as promising materials in energy storage and environmental remediation because of their unique physicochemical properties and low cost. However, the fabrication of multifunctional biochar materials with a well-developed hierarchical porous structure as well as self-doped functionalities via a facile strategy remains a challenge. Herein, we demonstrate a heteroatom-doped porous biochar, prepared by a hydrothermal pretreatment followed by a molten salt activation route. With the creation of a high specific surface area (1501.9 m^2^/g), a hierarchical porous structure, and the incorporation of oxygen-/nitrogen-functional groups, the as-prepared biochar (BC-24) exhibits great potential for supercapacitor application and organic pollutant elimination. The assembled biochar electrode delivers a specific capacitance of 378 F/g at 0.2 A/g with a good rate capability of 198 F/g at 10 A/g, and excellent cycling stability with 94.5% capacitance retention after 10,000 recycles. Moreover, BC-24 also exhibits superior catalytic activity for phenol degradation through peroxydisulfate (PDS) activation. The phenol (0.2 mM) can be effectively absorbed and then completely degraded within only 25 min over a wide pH range with low catalyst and PDS dosages. More importantly, TOC analysis indicates 81.7% of the phenol is mineralized within 60 min, confirming the effectiveness of the BC-24/PDS system. Quenching experiments and EPR measurements reveal that SO_4_·^−^ and ·OH as well as ^1^O_2_ are involved in the phenol degradation, while the non-radical pathway plays the dominant role. This study provides valuable insights into the preparation of cost-effective carbon materials for supercapacitor application and organic contaminant remediation.

## 1. Introduction

In recent years, due to the fast development of the global economy and the extensive depletion of fossil fuels, human society is facing increasingly severe problems including energy shortage and environmental contamination. Therefore, developing high-performance multifunctional materials for both energy and environmental applications has attracted significant research interest. Among all the strategies, the preparation of biochar from biomass waste has been considered as a sustainable and cost-effective route because of the unique merits of biomass, such as renewable sources, ease of availability, and low cost [1,2]. Moreover, biochars are widely studied as electrode materials or catalysts because of their high specific surface area (SSA), adjustable pore structure, abundant surface heteroatom dopants, and good electrical conductivity [3,4]. A variety of biomasses have been converted into biochar materials for applications including electrochemical supercapacitors [5,6,7,8], electrosynthesis [9,10], and environmental remediation [11,12,13].

For electrode material or catalyst usage, the SSA and pore structure have significant impacts on the performance of biochar products. Generally, micropores (<2 nm) are beneficial to increasing the available reaction sites, while mesopores (2–50 nm) ensure fast mass transportation of ions within the carbon skeleton by providing short ion diffusion channels. Therefore, an interconnected hierarchically porous framework is generally considered essential for the prepared biochar. Take the supercapacitor as an example, electronic energy is stored in a carbon electrode via two pathways, namely the electric double-layer capacitance and the faradaic pseudo-capacitance [14]. The presence of numerous reaction sites provided by microporosity enables a high specific capacitance, whereas mesopores can act as ion-migration channels, guaranteeing a high-rate capability. The pore structure of biochar can be tailored by changing the fabrication conditions. Among various preparation routes, an activation method is often employed, in which CO_2_, air, steam, etc. (physical activation) or KOH, K_2_CO_3_, ZnCl_2_, etc. (chemical activation) are commonly applied as activators thereby endowing higher porosity development [1,15,16]. For example, Wang et al. applied a KOH-assisted activation strategy to prepare bamboo-derived biochar with a developed porous structure—the constructed hybrid supercapacitor delivers an energy density of 114.2 Wh/kg with a power density of 800 W/kg [17]. However, the conventional activation process mainly takes place at the surface of the biomass matrix because of the undeveloped interior pore structure of the precursor, resulting in that the obtained biochar materials usually possess a high SSA but with too many narrow-sized micropores. As reported, for solvated electrolytic ions to penetrate pore texture to form an electric double layer at the electrode/electrolyte interface, pore diameters larger than 0.6–0.7 nm are often required [18,19]. Consequently, the activated carbon with an ultra-high SSA but mainly a narrow-sized microporous structure usually exhibits poor capacitive performance because of the electrochemical inaccessibility of these micropores. Moreover, the widely used activating agents would inevitably cause a severe equipment corrosion problem (KOH), a secondary pollution issue (ZnCl_2_), or need a higher activation temperature of 900 or 950 °C (K_2_CO_3_). Therefore, developing a sustainable method for the preparation of high-performance biochar materials is of great importance. Recently, hydrothermal carbonization has presented as a promising preprocessing method for biochar production, which creates an interior pore structure by hydrolyzing the unstable constituents in the biochar matrix [20,21]. More importantly, it is worthwhile mentioning that surface functionalities may be incorporated through carryover from the biomass precursors which are commonly rich in heteroatoms of O and N. As reported, surface functionalization is another important strategy of enhancing capacitive performance for supercapacitors. In particular, oxygen- or nitrogen-containing groups can not only increase the wettability of the biochar electrode through hydrophilic functional groups, but also improve the pseudo-capacitive performance via faradaic redox reactions [22,23,24].

Recently, persulfate-based advanced oxidation processes (AOPs) have emerged as promising technologies for the effective elimination of recalcitrant organic contaminants in wastewater, owing to the generation of powerful reactive oxygen species (ROS) including sulfate radical (SO_4_·^−^), hydroxyl radical (·OH), superoxide radical (O_2_^−^), and singlet oxygen (^1^O_2_) [25,26,27]. In comparison with the conventional AOP of Fenton reactions, persulfate-based AOP has a higher oxidation potential (2.5–3.1 V for SO_4_·^−^ vs. 1.8–2.7 V for ·OH), longer lifespan (t_1/2_ = 30–40 μs vs. t_1/2_ < 1 μs), and wider pH range applicability (pH = 2−8 vs. pH = 3) [28,29]. Nevertheless, persulfate (PS) relies on an activation process to obtain high oxidation capacity, such as thermal activation, UV irradiation, ultrasonication, carbon-based or metal/metal oxide activation [26,27,28]. Among these methods, the carbon-based activation systems have shown greater potential because of their advantages of strong adsorption capability, abundant surface functional groups, good electronic conductivity, and being free of secondary pollution [29,30]. Thereinto, biochar catalysts prepared from biomass waste with a hierarchically porous structure and doped, abundant heteroatoms have attracted tremendous attention. Of note, the carbon/biochar-PS heterogeneous catalytic system can degrade organic contaminants not only through the generation of reactive radicals but also via a nonradical (electron-transfer) pathway [31,32]. The sufficient micropores supply active sites to increase the adsorption capacity of pollutants, while mesopores allow fast mass transportation thereby improving the degradation efficiency. In addition, the synergistic effect facilitates PS activation encountered in the catalytic process with nitrogen- or oxygen-doped biochar [33,34,35]. As reported, nitrogen functionality can reconstruct the electron distribution of the neighboring carbon atoms thereby promoting catalytic activity toward PS activation by enhancing electron transfer ability [31,36]. In general, graphitic N can improve electrical conductivity and accelerate decomposition of PDS, leading to the generation of ^1^O_2_ and facilitating electron transfer; while pyridinic N can serve as an electron donor to organic pollutants and capture PDS molecules with high electrophilic characteristics [29]. Meanwhile, the oxygen functional groups can not only tailor the reductivity of the carbon framework but also regulate the zeta potential of the carbon surface, thereby affecting the interaction with the negatively charged persulfate ions [34]. Moreover, the ketonic/carbonyl groups (C=O) and carbonyl groups are considered as the primary active sites for ^1^O_2_ generation in PS activation [37], and can act as Lewis basic sites for PDS activation to generate superoxide radicals and/or hydroxyl radicals [38], while hydroxyl groups are favorable to the electron-transfer process because of their electron-donating characteristic [34].

In this study, a heteroatom-doped porous biochar was fabricated by a coupled hydrothermal pretreatment and molten salt activation process. The hydrolyzing of unstable compositions in biomass during the hydrothermal process leads to the formation of many interior pores in the resulting hydrochar. These preformed mesopores are vital for the generation of micropores and the creation of larger mesopores in the following molten salt activation step (Na_2_CO_3_-K_2_CO_3_ at 800 °C) since they permit better contact interaction with the molten agents, thereby developing a hierarchical pore texture of the prepared biochar materials. With the well-developed hierarchical porous structure as well as incorporated surface oxygen- and nitrogen dopants, the as-prepared biochar electrode exhibits a good potential in application as a supercapacitor. Moreover, the obtained biochar can also serve as an effective catalyst for phenol degradation through PDS activation.

## 2. Materials and Methods

### 2.1. Materials and Chemicals

The biomass, i.e., water hyacinth was collected from the South Lake in Wuhan. After washing and drying, the biomass was pulverized into fine powder through an 80 mesh sieve and stored for use. Sodium sulfate (Na_2_SO_4_), sulfuric acid (H_2_SO_4_), sodium carbonate (Na_2_CO_3_), potassium carbonate (K_2_CO_3_), potassium peroxydisulfate (PDS), potassium iodide (KI), methyl alcohol (MeOH), *tert*-butyl alcohol (TBA), and furfuryl alcohol (FFA) were purchased from Sinopharm Chemical Reagent Co., Ltd. (Shanghai, China). Polytetrafluoroethylene (PTFE, 60 wt%), phenol, 5,5-dimethyl-1-pyrroline N-oxide (DMPO), and 2,2,6,6-tetramethyl-4-piperidinol (TEMP) were obtained from Shanghai Aladdin Biochemical Technology Co., Ltd. (Shanghai, China). Methanol of HPLC grade was purchased from Sigma-Aldrich (Shanghai, China). Nafion solution (5 wt%) was supplied by Dupont Ltd. All chemicals and reagents were used as received without any additional purification. Ultrapure water was used in all experiments.

### 2.2. Preparation of Biochar Samples

The biochar samples were prepared by a hydrothermal treatment followed by a molten salt carbonization/activation process. In a typical procedure, 6.0 g biomass powder together with 60 mL ultrapure water were added into a hydrothermal reactor and heated at 200 °C for 12, 24, and 48 h. After filtering and drying, the resulting hydrochar was mixed with Na_2_CO_3_–K_2_CO_3_ in a mass ratio of 1:4. The mixture was placed in an alumina boat which was located in a tubular furnace. Then, the temperate was heated up to 800 °C with 5 °C /min and maintained for 1 h under an N_2_ atmosphere. Afterwards, the resulting products were washed with 3 M HCl and ultrapure water several times to remove the residual salts and then dried in a vacuum oven overnight at 80 °C. The obtained biochar samples were named BC-*x*, where *x* is the hydrothermal reaction time. For comparison, a biochar prepared under similar procedures but without hydrothermal pretreatment was labeled as AC. NC represents a control sample prepared by directly carbonizing the biomass precursor under an N_2_ atmosphere at 800 °C for 1 h, where no hydrothermal as well as activation processes were applied.

### 2.3. Materials Characterization

The morphology and microstructure of the samples were observed using a Hitachi SU8010 field emission scanning electron microscopy (SEM). Pore textures of the prepared biochar samples were determined by N_2_ adsorption–desorption isotherms at 77 K using an ASAP 2020 instrument. X-ray diffraction (XRD) patterns were performed on a Bruker D8 Advance diffractometer with Cu Kα radiation. Raman spectra were conducted on a laser confocal microscopy Raman spectrometer (DXR, Thermo Fisher, Waltham, MA, USA) at 532 nm. X-ray photoelectron spectroscopy (XPS) was recorded on a Multilab 2000 with Al Kα radiation for surface chemical species detection.

### 2.4. Electrochemical Measurements

The electrochemical measurements were conducted in a three-electrode system with 1 M H_2_SO_4_ as the electrolyte, where an assembled carbon film was applied as the working electrode, and a saturated calomel electrode (SCE) and a Pt plate were provided as the reference electrode and counter electrode, respectively. For the carbon film preparation, biochar products (80 wt%), acetylene black (10 wt%), and PTFE binder (10 wt%) were mixed in 5 mL ethanol under magnetic stirring for 6 h. After volatizing the alcohol, the resulting paste was rolled into thin film and pressed on a titanium mesh current collector with a mass loading of 2–3 mg/cm^2^. Electrochemical impedance spectroscopy (EIS) tests were carried out using a PARSTAT 4000A electrochemical workstation at a sweeping frequency range of 10^−3^ Hz to 10^5^ Hz. Cyclic voltammetry (CV), galvanostatic charge–discharge (GCD) and cycling tests were conducted to determine the capacitive performance of the biochar electrodes using a CHI 660E electrochemical workstation. The specific capacitance (*C_s_*, F/g) was calculated from the GCD tests according to: *C_s_* = *I*Δ*t*/*m*Δ*V*, where *I* (A), Δ*t* (s), *m* (g), and Δ*V* (V) are the discharge current, discharge time, mass loading of biochar, and the potential change between −0.40 and 0.60 V (vs. SCE), respectively [20,21]. For electron-transfer mechanism investigation, a chronoamperometry test (I-t curve) was performed at the open circuit potential with a biochar catalyst coated glassy carbon electrode (GCE) in 0.1 M Na_2_SO_4_ electrolyte. The preparation procedure of the GCE working electrode has been described elsewhere [10].

### 2.5. Catalytic Degradation Experiments

The degradation experiments were conducted in 100 mL conical flasks at 25 °C. For each test, 5 mg of the as-prepared biochar catalyst (0.1 g/L) was added into 50 mL of phenol solution (0.2 mM). After reaching the adsorption–desorption equilibrium (15 min), a specific concentration of PDS was added into the solution to initiate the catalytic reaction. At predetermined intervals, 1 mL aliquot was sampled from the flask, filtered through a 0.22 μm filter film, and injected into an HPLC vial with 0.5 mL methanol rapidly to quench the catalytic reaction. The initial pH was adjusted with 0.1 M H_2_SO_4_ and 0.1 M NaOH solution. The quenching experiments were carried out by adding radical scavengers of MeOH, TBA, FFA, and KI into the solution.

### 2.6. Analytical Methods

The concentration of phenol was analyzed using high performance liquid chromatography (HPLC, Ultimate 3000, Thermo Fisher, Waltham, MA, USA) with a DAD detector at the wavelength of 270 nm and a reversed-phase column (Phenomenex, Torrance, CA, USA, C18, 5 µm, 4.6 × 250 mm). The mobile phase was composed of methanol and water (70:30, *v/v*) at a flow rate of 1.0 mL/min, an injection volume of 20 μL, and a column temperature of 30 °C. Electron paramagnetic resonance (EPR) analysis was conducted using a Bruker EMXnano spectrometer to identify the generated reactive species. DMPO was applied as the spin-trapping agent for SO_4_·^−^ and ·OH, and TEMP was selected as the spin-trapping agent for detecting ^1^O_2_. Total organic carbon (TOC) analysis was determined using a Multi N/C 3100 TOC analyzer.

## 3. Results and Discussion

### 3.1. Morphological and Structural Characterization

The morphology and porous architecture play an essential role in the capacitive performance as well as the adsorption capacity and catalytic activity of the prepared biochar materials since they are highly associated with the provided reaction sites and mass-transfer process. Herein, a hydrothermal process was employed to remove the unstable constituents (i.e., hemicellulose, cellulose, and other low molecular weight saccharides) in the biomass precursor and to widen the interior pore diameter of the resulting hydrochar [20,21]. Then, a molten salt treatment followed to further carbonize/activate the hydrochar samples, enrich the porosity, and to obtain hierarchically porous biochar [39].

**Figure 1:**
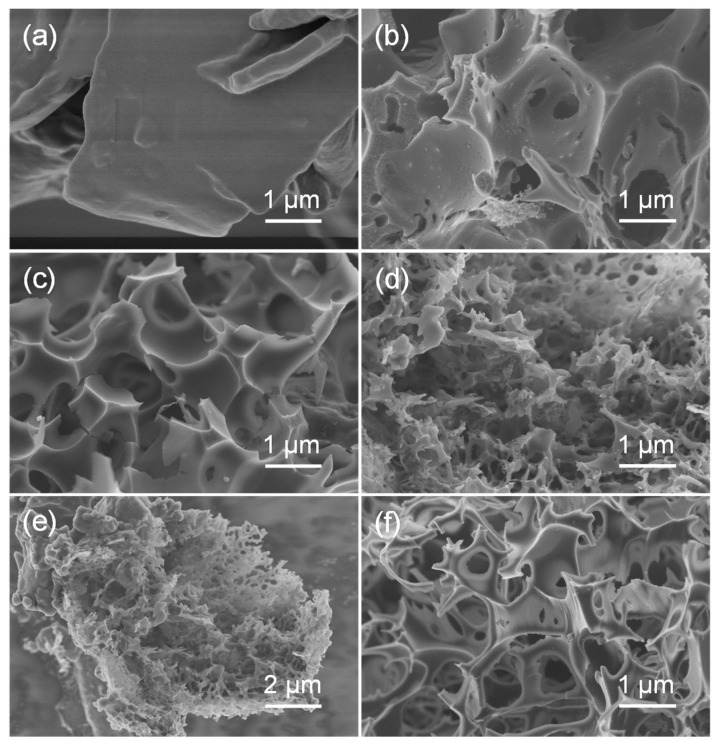
SEM images of NC (**a**), AC (**b**), BC-12 (**c**), BC-24 (**d**,**e**), BC-48 (**f**).

As the morphology observations of SEM showed, the NC sample prepared through direct carbonization of the biomass precursor under an N_2_ atmosphere displays a carbon sheet with a smooth surface (Figure 1a). When a molten salt activation step was applied, a more porous morphology with an inter-connected pore structure was obtained (AC, Figure 1b). For the BC-*x* samples, finer porous characteristics with a rough and uneven surface were observed, which could be attributed to the hydrolysis of unstable components in the biomass during the hydrothermal pretreatment. Moreover, with the hydrothermal reaction time prolonged from 12 to 24 h, more degradable compositions were hydrolyzed, resulting in the creation of a hierarchical porous structure of the prepared BC-24 (Figure 1d,e). Furthermore, EDX elemental mapping images presented in Figure S1 displayed uniform dispersion of O and N elements in the biochar skeleton. However, a longer treatment time of 48 h may have caused excessive hydrolyzation [21], leading to the collapse of pore texture and loss of microporosity (Figure 1f).

**Figure 2:**
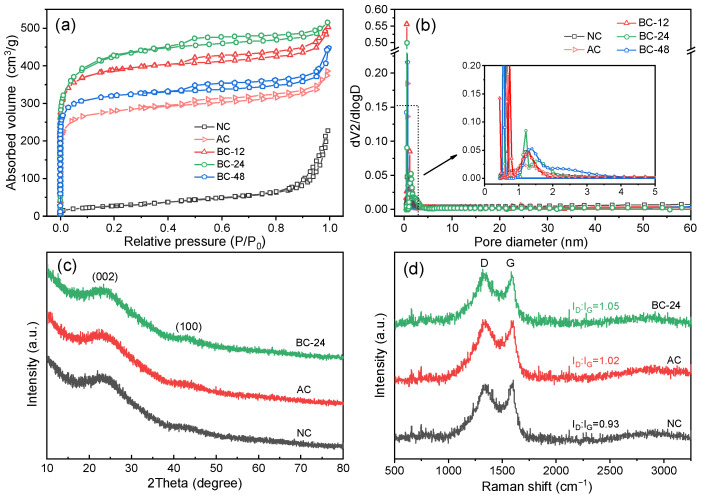
(**a**) N_2_ adsorption-desorption isotherms, (**b**) pore size distribution curves, (**c**) XRD patterns, and (**d**) Raman spectra of the indicated biochar samples.

Nitrogen adsorption–desorption isotherms of AC and BC-*x* in Figure 2a display typical type IV adsorption curves according to the classification by IUPAC [40]. The steep increment of nitrogen adsorption at a low relative pressure (P/P_0_ = 0.01) suggests the generation of micropores in large quantity when a molten salt activation step was employed. The resulting AC exhibits a specific surface area (SSA) of 971.4 m^2^/g, which is 9.7 times that of NC (Table 1). Moreover, the higher absorption volumes and more distinct hysteresis loops located at the relative pressure of 0.45 to 1.0 indicate that a large number of micro- and meso-pores were formed on the biochar products of BC-*x*, resulting in higher SSA and pore volumes. The formed micropores can provide numerous reaction sites for charge storage and organic pollutant adsorption, while mesopores can serve as channels for facilitating the mass transfer process. However, for BC-48, the decreased nitrogen uptake confirmed the collapse of the microporous structure because of excessive hydrolyzation, giving rise to a lower SSA of 1118 m^2^/g than that of BC-24 (1501.9 m^2^/g). The pore size distribution curves manifest that the generated pores are mainly micropores smaller than 2 nm (Figure 2b). These results are very consistent with the SEM observation and indicate that both the hydrothermal pretreatment and molten salt activation process are conducive to enriching the porous structure and creating active sites in the obtained biochar. Specifically, BC-24 possessed the highest SSA and the largest pore volume (1.14 cm^3^/g), demonstrating its good application potential.

The XRD patterns exhibit amorphous crystalline phases for all three biochar samples with two broad diffraction peaks at 2θ of 23.4° and 43.2° (Figure 2c), which could be allocated to the (002) and (100) planes of disordered carbon layer formed by crystal defects [21]. In addition, Raman spectra of the synthesized biochars shown in Figure 2d are reflected by two characteristic peaks located at 1340 cm^−1^ (D band) and 1580 cm^−1^ (G band). The D band could be assigned to the disorder and defects in the carbon layer, while the G band originates from the crystalline phases and graphitic structures. Therefore, the intensity ratio of D band to G band (I_D_/I_G_) can be used to estimate the defective degree of the biochar materials. The I_D_/I_G_ values for NC, AC, and BC-24 are 0.93, 1.02, and 1.05, respectively, suggesting that both the hydrothermal treatment and molten salt activation process helped in the creation of defective sites in the biochar matrices. The formation of defective edges in the biochar networks are favorable for improving the catalytic activity of biochars as catalysts [41].

**Figure 3:**
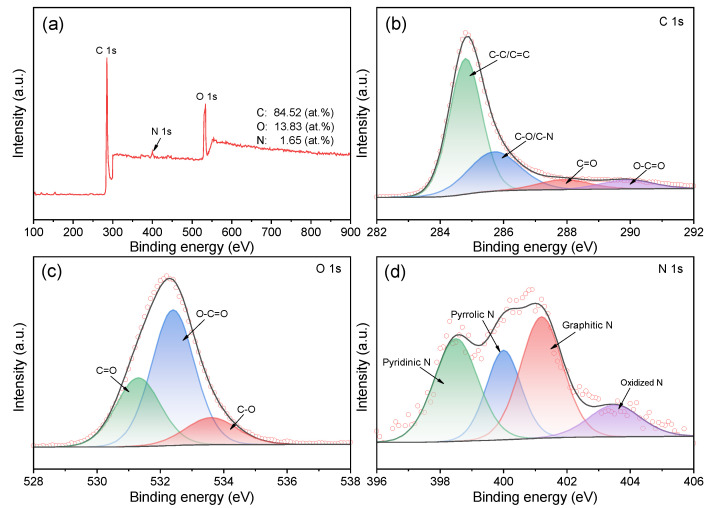
(**a**) XPS full survey spectrum and the high resolution deconvoluted (**b**) C 1s, (**c**) O 1s, and (**d**) N 1s spectra for BC-24.

XPS analysis was then conducted to evaluate the surface chemical compositions of the as-prepared BC-24, and three characteristic peaks assigned to C 1s (284.8 eV), O 1s (532.6 eV), and N 1s (400.1 eV) were observed (Figure 3a). The atomic percentages of O and N are 13.83 at% and 1.65 at%, respectively, further confirming the successful doping of oxygen- and nitrogen-functional groups in the biochar skeleton. The high-resolution C 1s spectrum shown in Figure 2b can be identified as containing C–C or C = C (284.8 eV), C–O or C–N (285.7 eV), C=O (287.9 eV), and O–C=O (290.0 eV) [42]. The O 1s spectrum (Figure 2c) can be deconvoluted into three regions with binding energies at 531.3, 532.4, and 533.6 eV, which originates from the carbonyl (C=O), carboxyl (O–C=O), and hydroxyl group (C–O) [34]. As reported, the carbonyl groups can serve as basic Lewis sites to activate persulfates by producing free radicals or initiating singlet oxygenation, while the hydroxyl groups are favorable for the electron-transfer process because of their electron-donating characteristic [34,43,44]. In Figure 2d, the N 1s spectrum was fitted into four peaks at 398.4 eV (pyridinic N), 400.0 eV (pyrrolic N), 401.1 eV (graphitic N), and 403.4 eV (oxidized N) [45]. The nitrogen dopants typically acknowledged can tailor the electronic mobility of the neighboring carbon lattice by modifying the electron density. More importantly, these incorporated oxygen- and nitrogen-functional groups may improve the wettability of the biochar electrodes in aqueous electrolyte and participate in the faradaic reactions that enhance the pseudo-capacitance of the biochar-based supercapacitor [46,47].

### 3.2. Electrochemical Measurements

**Figure 4:**
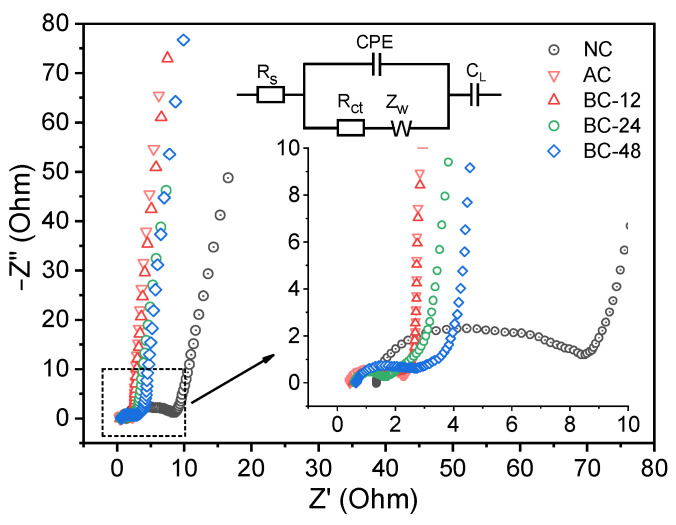
Nyquist plots of the indicated biochar electrodes.

To estimate the electronic conductivity and electron/ion transfer process of the biochar electrodes, EIS tests were then undertaken. As shown in Figure 4, the Nyquist plots of all electrodes consist of two semicircles. The intersect with the *x*-axis refers to the internal resistance (R_s_), which is correlated with the combined resistance of the electrode material and electrolyte [48]. According to the fitted Nyquist plots (Figure S2) and the corresponding equivalent circuit diagram (inset of Figure 4), the R_s_ values for NC, AC, BC-12, BC-24, and BC-48 are 1.31, 0.44, 0.56, 0.61, and 0.66 Ω (Table S1), respectively, signifying negligible intrinsic resistance and excellent electronic conductivity of the biochar electrodes. Meanwhile, the semicircle loops located at the high-frequency range are related to the charge transfer resistance (R_ct_), which should arise from the electronic and ionic resistance at the interface of electrode and electrolyte [49]. The R_ct_ values for NC, AC, BC-12, BC-24, and BC-48 are 5.93, 1.67, 1.10, 0.82, and 1.61 Ω, respectively. Among these electrodes, BC-*x* and AC have much smaller R_ct_ compared with that of NC, and BC-24 possesses the minimal one, indicating its superior electron/charge transfer efficiency. The decreased resistance could be owing to the optimized pore structure of BC-24, thus improving the electronic conductivity and electron transportation of the electrode. More importantly, all electrodes exhibited negligible Warburg resistance (R_w_) of 0.42–0.46 Ω (Table S1), indicating facilitated diffusion/transportation of electrolyte ions into the electrode materials [50]. In addition, the BC-*x* and AC exhibited nearly vertical slopes at low frequency range, implying the ideal capacitive behaviors of these electrodes.

**Figure 5:**
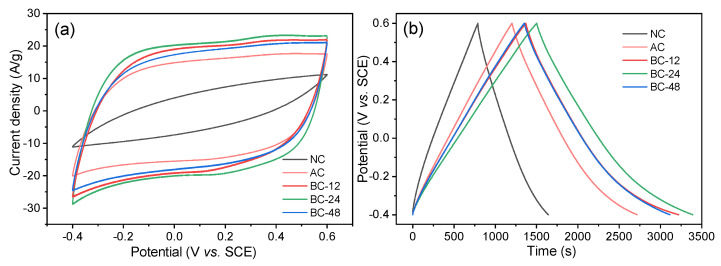
(**a**) CV curves at 100 mV/s and (**b**) GCD tests at 0.2 A/g for the different biochar electrodes.

To further evaluate the capacitive performance of the prepared biochar products, CV and GCD measurements were conducted. As shown in Figure 5, NC demonstrated an olive-like shaped CV curve while those for AC and BC-*x* maintained quasi-rectangular profiles at a scan rate of 100 mV/s. In particular, the curve of BC-24 exhibited the largest integral area with the same mass loading of active material in one electrode, suggesting the improved capacitive performance. Moreover, GCD curves display a longer discharge time for BC-*x* than those of AC and NC. The specific capacitance (*C_s_*, F/g) can be calculated from the discharge parts of the GCD curves. At a current density of 0.2 A/g, the *C_s_* values for NC, AC, BC-12, BC-24, and BC-48 were calculated to be 170, 303, 370, 378, and 352 F/g, respectively. The enhanced capacitive performance of AC and BC-*x* could be mainly ascribed to the enriched numerous reaction sites resulting from the generation of microporous structures during the preparation process. However, BC-48 exhibited smaller integral area and decreased discharge time than those of BC-24, indicating the overtreated hydrothermal process caused the collapse of the internal pore texture and resulted in the loss of microporous sites.

**Figure 6:**
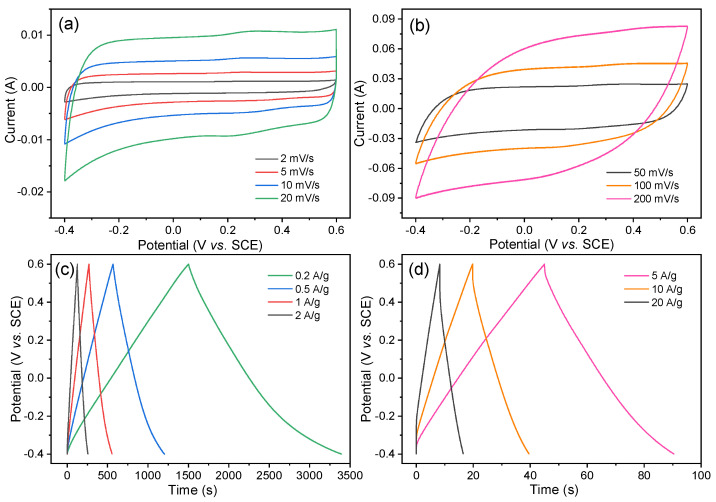
(**a**,**b**) CV profiles at different scan rates and (**c**,**d**) GCD curves at different current densities for BC-24.

The capacitive performance of BC-24 was then investigated by CV and GCD tests with different scan rates and current densities. As depicted in Figure 6, the CV curves exhibit quasi-rectangular shapes at all scan rates and the profile is well maintained even at a high scan rate of 200 mV/s (Figure 6b). Moreover, all GCD plots show triangular symmetric profiles and no distinct IR drops are observed at high current densities (Figure 6d), which could be attributed to the negligible internal resistance of BC-24. These results both indicate the highly capacitive feature of BC-24 with fast ion response. The presence of small bumps in the CVs (Figure 6a) and the carryover effect in the discharge parts of the GCD curves (Figure 6c) at a potential range of −0.20 to −0.40 V suggest that some redox reactions were involved, which could be correlated with the pseudo-capacitance resulting from the electron transfer of heteroatom-doped surface functional groups [51].

**Figure 7:**
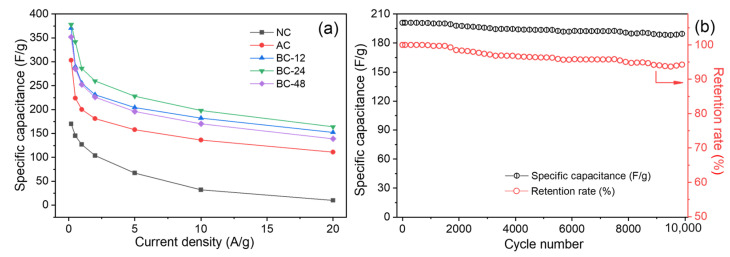
(**a**) Comparison of the gravimetric capacitive performance of the indicated biochar electrodes at different current densities. (**b**) Cycling stability test of BC-24 at 10 A/g.

Based on the GCD curves, the gravimetric specific capacitances at different current densities were calculated and BC-24 exhibited the best capacitive performance in comparison with the other investigated electrodes (Figure 7a). The *C_s_* values of BC-24 are 378, 342, 286, 260, 228, 198, and 164 F/g at current densities of 0.2, 0.5, 1, 2, 5, 10, and 20 A/g, respectively, which are superior to the reported biochar-based supercapacitors (Table S2). The improved capacitive performance of BC-24 could be ascribed to generation of more microporosity, providing numerous active sites for charge storage (electric double-layer capacitance), as well as due to the presence of nitrogen- and oxygen-functional groups which create greater capacitance though faradaic reactions (pseudo-capacitance). Moreover, the formation of mesopores shortened the ion diffusion and migration pathway, thereby promoting the fast charge–discharge process. To evaluate the long-term stability of the biochar electrode, the cycling performance of BC-24 was recorded using a GCD test with a current density of 10 A/g. As shown in Figure 7b, the specific capacitance decreases from 200 F/g to 189 F/g after 10,000 cycles with a high retention rate of 94.5%, demonstrating excellent cyclic stability sustained by the hierarchical porous structure of BC-24.

### 3.3. Catalytic Activity for Phenol Degradation through PDS Activation

**Figure 8:**
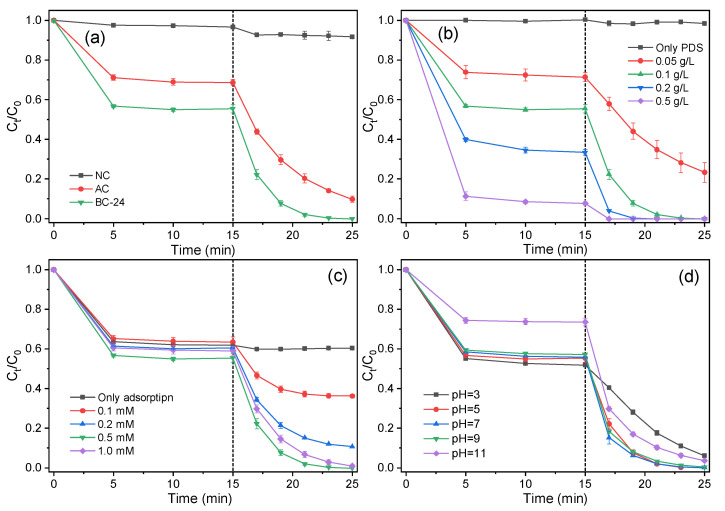
Effects of biochar catalysts (**a**), catalyst dosage (**b**), PDS concentration (**c**), and initial pH (**d**) on phenol degradation ([phenol] = 0.2 mM, catalyst dosage = 0.1 g/L, [PDS] = 0.5 mM).

To further explore the catalytic activity of the as-prepared biochar products toward PDS activation for organic pollutant elimination, phenol was selected as the target contaminant. Before the degradation, biochar samples were dispersed into a phenol solution and oscillated for 15 min to achieve adsorption–desorption equilibrium. Then, a certain amount of PDS was added to trigger the catalytic reactions. As depicted in Figure 8a, only 3.4% of phenol was absorbed during the pre-adsorption process for NC, while the removal for AC and BC-24 reached 31.3% and 44.6%, respectively. The higher adsorption capacity of BC-24 could be mainly ascribed to the higher specific surface area and enriched microporosity, which provide sufficient adsorption sites for phenol removal. After the addition of PDS, only 8.3% of the original phenol was removed for NC in 10 min, suggesting poor degradation efficiency caused by limited reactive sites of NC (100.1 m^2^/g, Table 1). However, for the biochar catalyst prepared with activation procedure (AC), the removal increased dramatically to 98.5% after reacting for 10 min, suggesting the higher SSA (971.4 m^2^/g) not only improved the adsorption capacity but also promoted the catalytic degradation process. For BC-24, 0.2 mM of phenol was completely degraded within 10 min. The degradation curves fitted well with the pseudo-first-order reaction kinetics, and the kinetic constants (*k*) of NC, AC, and BC-24 were calculated to be 0.011, 0.249, and 0.683 min^−1^, respectively. Besides the hierarchical porous structure which can promote degradation efficiency by providing numerous active sites and facilitating the charge/mass transfer process, the heteroatom dopants may also contribute substantially to removal efficiency. For example, Guo et al. reported that the incorporation of edge nitrogen dopants (pyridinic N and pyrrolic N) can serve as reactive sites for persulfate activation [52]; while graphitic N can tailor the electronic property of the neighboring carbon lattice and facilitate electron transfer from organic contaminants to the PDS/carbon complexes, thereby accelerating the catalytic degradation reactions through a nonradical pathway [45]. Meanwhile, C=O functional groups (carbonyl groups in quinone and pyrone) at the defective edges are reported to act as Lewis basic sites for PDS activation to generate free radicals [38].

The effect of biochar dosage on phenol removal was investigated and the degradation efficiencies were positively correlated with the amount of added catalysts. As displayed in Figure 8b, the sole PDS system exhibited negligible contribution for phenol removal. However, when 0.05 g/L biochar catalysts (BC-24) were added, 28.6% of phenol was removed under adsorption for 15 min and 76.6% was degraded after reaction for 10 min, demonstrating the important role of biochar catalysts in phenol degradation. After increasing the catalyst dosage to 0.1 g/L, 0.2 mM of phenol was 100% removed within only 25 min and the kinetic constant increased from 0.161 min^−1^ (0.05 g/L) to 0.683 min^−1^. When further increasing the biochar dosages to 0.2 and 0.5 g/L, the removal of phenol via pre-adsorption reached 66.5% and 92.3%, respectively, indicating more active sites were supplied with higher catalyst dosages. Considering the economic aspects of the process, a catalyst dosage of 0.1 g/L was selected for the following experiments. The effect of PDS concentration on the catalytic performance of BC-24 towards phenol degradation is shown in Figure 8c, and no obvious catalytic removal was observed for the system without the addition of PDS. When increasing the concentration of PDS from 0.1 to 0.5 mM, complete removal was achieved with 10 min of reaction, and the kinetic constant increased from 0.134 min^−1^ to 0.683 min^−1^. However, further increase of PDS dosage (1.0 mM) resulted in deteriorated degradation efficiency (*k* = 0.457 min^−1^), which could be ascribed to the self-quenching effect caused by the presence of excessive PDS in the system [53]. It is noteworthy to mention that, compared with the wildly adopted carbon-based catalyst dosages of 0.5–1.0 g/L and PDS concentration of 4–6 mM in previous reports [36,42,53], the lower catalyst (0.1 g/L) and PDS (0.5 mM) amounts needed in this work undoubtedly signify better catalytic performance and lower economic costs. To explore the effect of initial pH on phenol removal, degradation experiments were conducted at a pH range of 3–11. As presented in Figure 8d, effective degradation can be achieved over a wide pH range, indicating good catalytic capability and remarkable applicability of BC-24. Notably, the adsorption of phenol was heavily inhibited at pH 11, which can be attributed to the electrostatic repulsion effect between phenol and biochar catalysts under basic conditions [54]. However, despite the depressed adsorption efficiency, a satisfactory catalytic reaction kinetic was still achieved (*k* = 0.355 min^−1^) at pH 11, which could be because of the alkaline activation of PDS under strong alkaline conditions [55,56].

**Figure 9:**
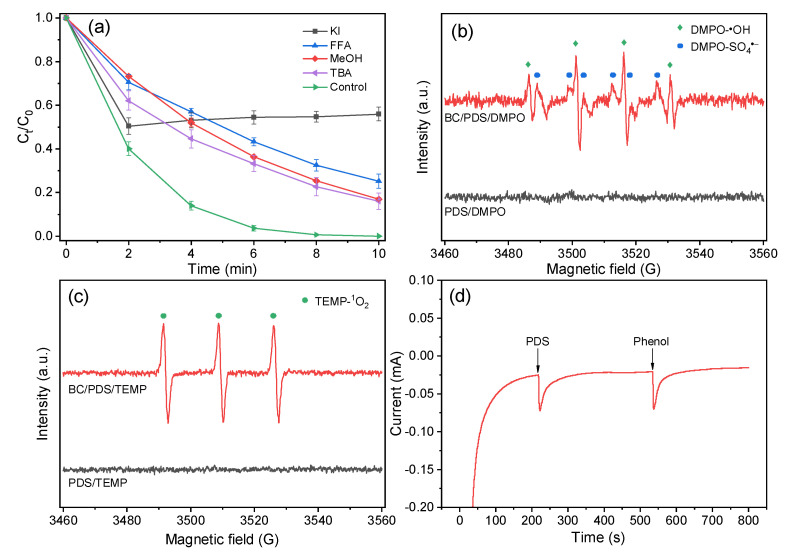
(**a**) Quenching effect on phenol degradation of BC-24 during PDS activation by different scavengers (catalyst dosage = 0.1 g/L, [PDS] = 0.5 mM, [phenol] = 0.2 mM, [MeOH] = [TBA] = [FFA] = [KI] = 10 mM). EPR spectra of PDS activation by BC-24 with the addition of (**b**) DMPO and (**c**) TEMP as spin-trapping agents. (**d**) Chronoamperometry test with the addition of PDS and phenol.

**Figure 10:**
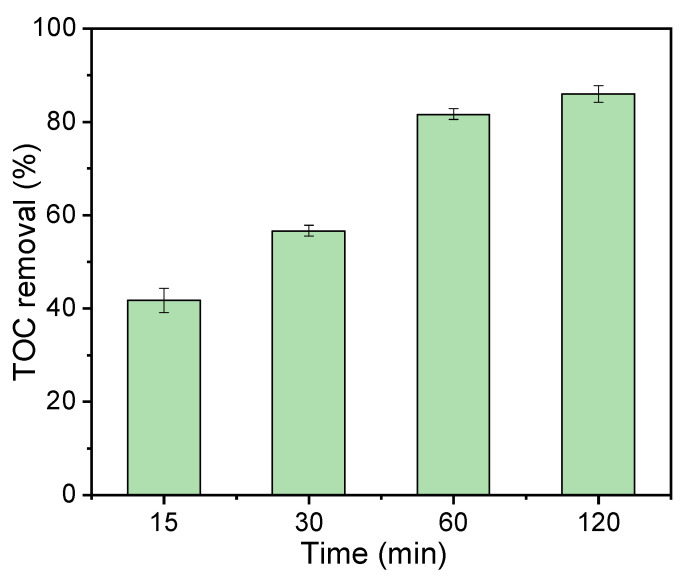
TOC removal for phenol mineralization by BC-24/PDS system.

The catalytic reaction mechanism of the degradation process was investigated by quenching experiments, where MeOH was employed as a scavenger for both SO_4_·^−^ and ·OH, TBA for ·OH, and FFA for ^1^O_2_. As the results show in Figure 9a, insignificant inhibiting impact was observed with the addition of 10 mM MeOH or TBA into the solution, revealing that free radicals did not play a major role in the BC-24/PDS system for phenol degradation. In comparison, FFA exhibited a slightly higher quenching ability than MeOH and TBA, inferring the generation of ^1^O_2_ provided an important contribution in phenol removal. KI was then applied to quench the surface-bound reactive oxygen species, and 49.6% of phenol was removed within the first two minutes, which could be ascribed to the unique adsorption capability of the biochar catalysts. However, the catalytic reaction was completely terminated, and the residual concentration of phenol was increased to some degree because of the desorption process, suggesting that the surface-bound (BC-24/PDS) complexes played a crucial role in phenol degradation [45,52]. These results indicate that both radical and nonradical pathways contributed to the catalytic degradation of phenol while the nonradical pathway played the dominant role. EPR measurements were then employed to further probe the generation of reactive species in the BC-24/PDS system, where DPMO and TEMP were applied as spin tripping agents. As shown in Figure 9b, characteristic signals ascribed to DMPO-·OH and DMPO-SO_4_·^−^ adducts were detected, revealing the generation of hydroxyl and sulfate radicals [30,57,58]. Moreover, the typical peaks of TEMP-^1^O_2_ adduct were observed (Figure 9c), confirming the presence of ^1^O_2_ [59]. To gain more evidence about the non-radical pathway for phenol degradation, a chronoamperometry test was conducted and the changes in current response with the addition of PDS or phenol were recorded. As presented in Figure 9d, notable current responses were detected with the injection of PDS and phenol, evidencing the occurrence of electron transfer among the biochar catalysts, PDS molecules, and phenol [52,59]. The good electrical conductivity (EIS in Figure 4) and edge nitrogenation (Figure 3d) of the biochar catalyst ensure fast electron transfer efficacy to form metastable reactive complexes at the PDS/biochar surface, which mainly account for the effective degradation of phenol. Moreover, the BC-24/PDS system also exhibits satisfactory catalytic phenol degradation performance in comparison with the previous reports with relatively lower catalyst and PDS dosages (Table S3). More importantly, TOC analyses indicate 81.7% and 86% of the original phenol were mineralized to release CO_2_ within 60 and 120 min, respectively, further confirming the effectiveness of the BC-24/PDS system (Figure 10). Unfortunately, the as-prepared biochar catalyst exhibited unsatisfactory reusability. As shown in Figure S3, the phenol removal decreased to 84.9% and 28.2% within 60 min in the second and third runs, respectively. The deactivation of catalyst could be ascribed to the coverage of intermediates on BC-24, which prevented the interaction of PDS with the active sites on BC-24 catalyst, thus inhibiting the electron-transfer process and limiting the production of reactive oxidizing species [60].

**Figure 11:**
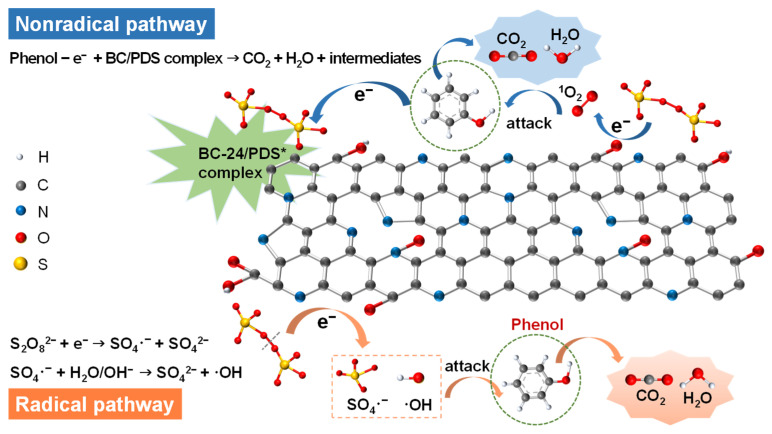
Proposed mechanism of phenol degradation through PDS activation on BC-24 catalyst.

According to the EPR analyses and chronoamperometry test results, the catalytic mechanism of phenol degradation in BC-24/PDS system was proposed, as shown in Figure 11. In the dominated nonradical pathway, the surface doping of pyridinic N and pyrrolic N greatly modulated the electron distribution of BC-24, thereby generating more reactive sites and facilitating the interaction between BC-24 and PDS to form a surface-bound complex of BC-24/PDS. In addition, the mediator role of BC-24 and the electron-donating characteristic of hydroxyl groups enhanced the electron-transfer process from phenol to PDS, thus resulting in the oxidation of the phenol due to the high redox potential of the BC-24/PDS complex [52]. Moreover, the important active sites of the ketonic/carbonyl groups (C=O) [37] as well as the improved electric conductivity through doping of graphitic N, promoted the generation of ^1^O_2_ [29], thus resulting in the degradation of phenol. Meanwhile, the C=O/O–C=O functional groups at the defective edges can act as Lewis basic sites for PDS activation to generate free radicals of SO_4_·^−^ and ·OH, thereby contributing to the radical pathway for phenol remediation [34,38,43,44].

## 4. Conclusions

A porous biochar with heteroatom dopants was synthesized via a hybrid hydrothermal pretreatment and molten salt activation strategy. With the generated hierarchical porous structure as well as incorporated oxygen- and nitrogen-containing functional groups, the obtained biochar exhibits excellent electrochemical properties. When applied as an electrode material for a supercapacitor, the as-prepared BC-24 shows high specific capacitance, fast charge–discharge rate capability, and good long-term stability. Moreover, BC- 24 also presents superior catalytic activity for phenol degradation through PDS activation. The phenol can be effectively absorbed and then degraded within only 25 min over a wide pH range with low catalyst and PDS dosages. Both the radical pathways (sulfate and hydroxyl radicals) and the non-radical pathway contribute to the removal of phenol, where the latter one plays a predominant role. This study provides important insights into the fabrication of cost-affordable biochar materials for energy storage application and organic contaminant elimination.

## Figures and Tables

**Table 1:** Porosity analysis of NC, AC, and BC-*x*.

Samples	S_BET_ (m^2^/g)	Total Pore Volume (cm^3^/g)	Micropore Volume (cm^3^/g)	Proportion of Mesopore (%)	Average Pore Size (nm)
NC	100.1	0.34	0.33	2.79	13.10
AC	971.4	0.96	0.59	38.78	2.42
BC-12	1362.2	1.13	0.76	32.71	2.23
BC-24	1501.9	1.14	0.80	30.08	2.12
BC-48	1118.0	0.88	0.66	25.79	2.35

## Data Availability

The data presented in this study is available on request from the corresponding author.

## Supplementary Materials

The following supporting information can be downloaded at: https://www.mdpi.com/article/10.3390/nano12152586/s1, Figure S1: EDX elemental mapping images of BC-24; Figure S2: Experimental and simulated Nyquist plots for the indicated electrodes; Figure S3: Cycling experiments for phenol degradation by the BC-24/PDS system; Table S1: The resistance parameters deduced from the equivalent circuits of EIS measurements; Table S2: Comparison of the capacitive performance of BC-24 in a three-electrode system with other biochar materials in the reported literature [6,20,61,62,63,64,65,66,67]; Table S3: Comparison of the catalytic phenol degradation performance of BC-24 through persulfate activation with reported literature [45,54,60,68,69,70,71,72].
