# Supplementary material for: Heteroatom-Doped Hierarchically Porous Biochar for Supercapacitor Application and Phenol Pollutant Remediation
Source: Nanomaterials (Basel). 2022 Jul 28;12(15):2586. doi: 10.3390/nano12152586 (PMC9370815; doi:10.3390/nano12152586)
Supplement: Supplementary file 1 [file nanomaterials-12-02586-s001.zip › nanomaterials-1829024-supplementary.pdf]

nanomaterials

Supplementary material

Heteroatom-Doped Hierarchically Porous Biochar for Supercapacitor Application and Phenol Pollutant Remediation

Diyong Tang [1,*], Li Lu [1], Zhipeng Luo [1], Baokun Yang [1], Jun Ke [2],Weidong Lei [1], Hongran Zhen [1], Yuan Zhuang [3], Jie Sun [1], Ke Chen [1] and Jie Sun [1]

[1] Key Laboratory of Resources Conversion and Pollution Control of the State Ethnic Affairs Commission, College of Resources and Environmental Science, South-Central Minzu University, Wuhan 430074, China; 2021110590@mail.scuec.edu.cn (L.L.); 2020120750@mail.scuec.edu.cn (Z.L.); 2020110555@mail.scuec.edu.cn (B.Y.); 2021120875@mail.scuec.edu.cn (W.L.); 202021241174@mail.scuec.edu.cn (H.Z.); 3047207@mail.scuec.edu.cn (J.S.); kechen@mail.scuec.edu.cn (K.C.); jetsun@mail.scuec.edu.cn (J.S.)

[2] School of Chemistry and Environmental Engineering, Wuhan Institute of Technology, Wuhan 430073, China; jke@wit.edu.cn

[3] Experimental Teaching and Engineering Training Center, South-Central Minzu University, Wuhan 430074, China; 8800319@mail.scuec.edu.cn

* Correspondence: tangdy076@mail.scuec.edu.cn

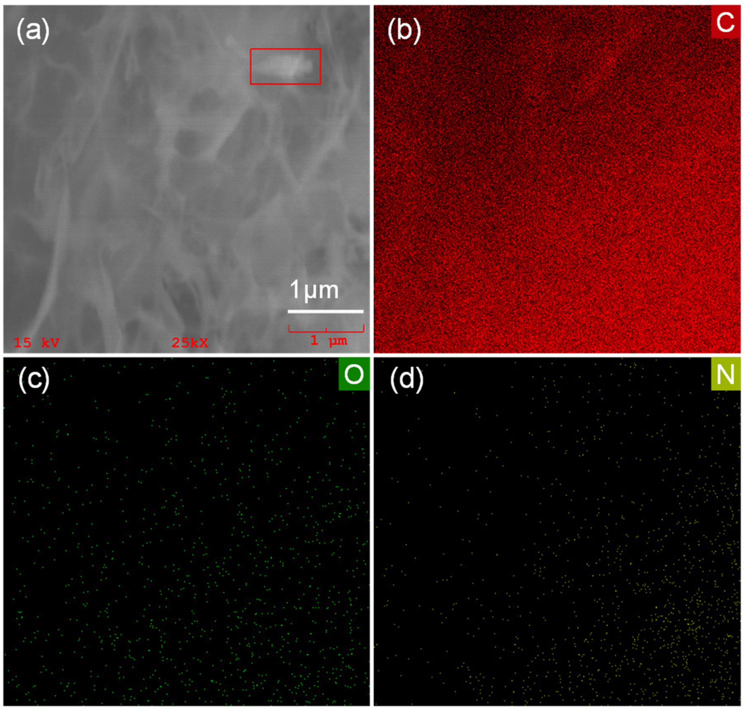

Figure S1. EDX elemental mapping images of BC-24.

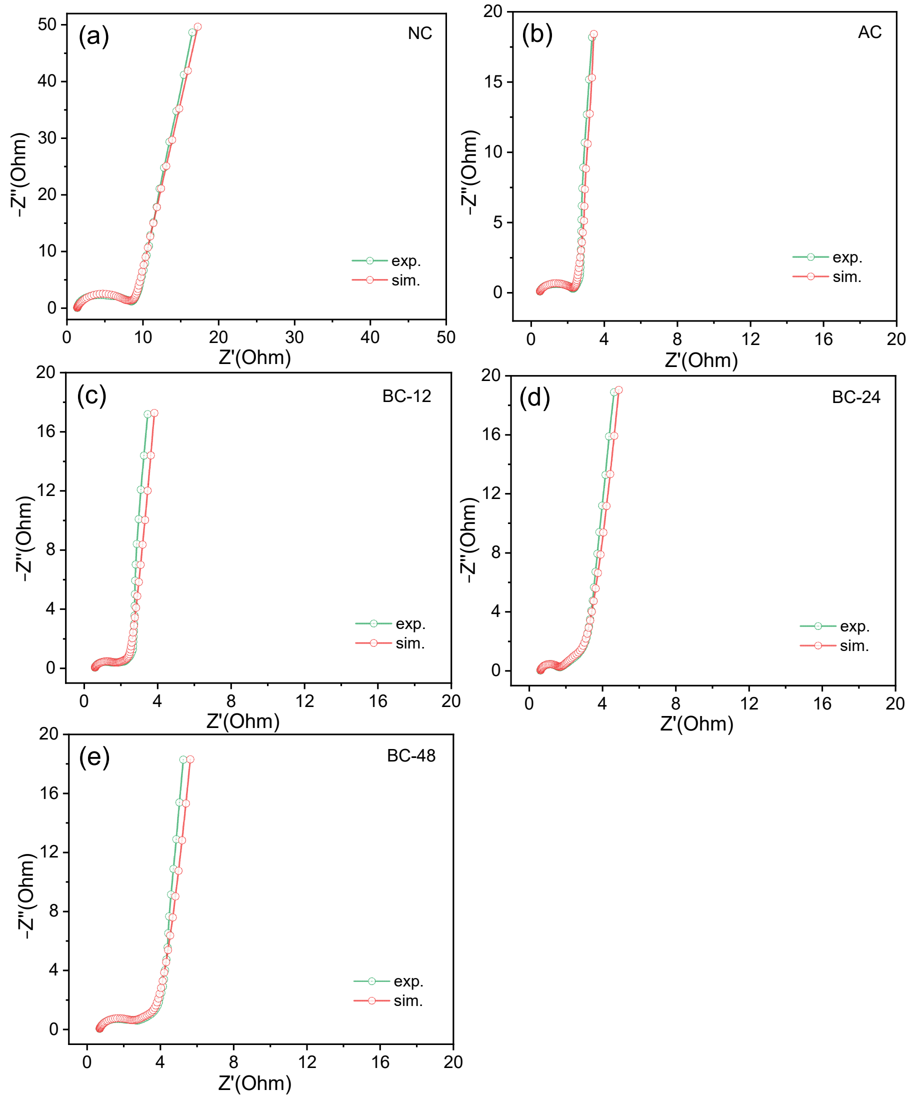

Figure S2. Experimental and simulated Nyquist plots for the indicated electrodes.

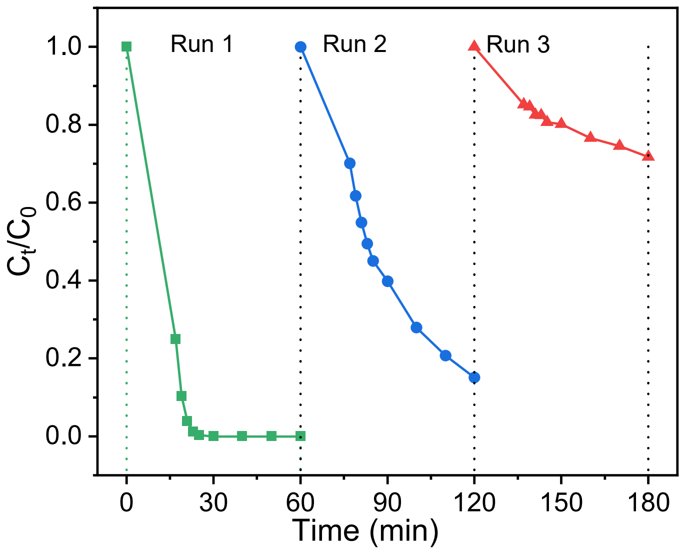

Figure S3. Cycling experiments for phenol degradation by the BC-24/PDS system ([phenol] = 0.2 mM, catalyst dosage = 0.1 g/L, [PDS] = 0.5 mM).

Table S1. The resistance parameters deduced from the equivalent circuits of EIS measurements.

**Electrodes**	**R_s (Ω)**	**R_{ct} (Ω)**	**R_w (Ω)**
NC	1.31	5.93	0.44
AC	0.44	1.67	0.44
BC-12	0.56	1.10	0.46
BC-24	0.61	0.82	0.46
BC-48	0.66	1.61	0.42

Table S2. Comparison of the capacitive performance of BC-24 in a three-electrode system with other biochar materials in the reported literature.

**Samples**	**Electrolyte**	**Current Density (A/g)**	**Gravimetric Specific Capacitance (F/g)**	**Ref.**
BBC-4	1 M H_2SO_4	0.3	259	[1]
PC-900	1 M H_2SO_4	0.5	169.4	[2]
C_K/H_3PO_4	1 M H_2SO_4	0.5	186.3	[3]
ACRP-650	1 M H_2SO_4	0.5	289	[4]
HM-24	1 M H_2SO_4	0.5	314	[5]
AA-RSC	1 M H_2SO_4	0.5	332	[6]
BC-24	1 M H_2SO_4	0.5	342	This work
S-AC	1 M KOH	10	85	[7]
AC_{TS}-1.0	6 M KOH	10	150	[8]
GHPB-0.2	6 M KOH	10	160	[9]
GHPB-0.2	1 M H_2SO_4	10	118	[9]
ACRP-650	1 M H_2SO_4	10	187	[4]
AA-RSC	1 M H_2SO_4	10	190	[6]
BC-24	1 M H_2SO_4	10	198	This work

Table S3. Comparison of the catalytic phenol degradation performance of BC-24 through persulfate activation with reported literature.

Catalysts	Experimental Conditions	Reaction Time (min)	Degradation Efficiency	k (min^{-1})	Stability	Ref.
AC950	[catalyst] = 0.2 g/L, [PS] = 5 mM, [phenol] = 0.5 mM	60	99.5%	0.087	Removal 10% after 3 cycles	[10]
Ca/BS-800-KOH	[catalyst] = 0.066 g/L, [PS] = 1 g/L, [phenol] = 20 mg/L	90	100%	0.040	Removal 48% after 3 cycles	[11]
GK-BC-850	[catalyst] = 0.5 g/L, [PS] = 1 mM, [phenol] =10 mg/L	60	90%	0.155	Removal 50% after 5 cycles	[12]
BM-α-FeOOH/PBC700	[catalyst] = 0.2 g/L, [PS] = 8.4 mM, [phenol] =50 mg/L	180	89.6%	0.100	Removal 82.9% after 4 cycles	[13]
BC	[catalyst] = 0.5 g/L, [PS] = 0.8 mM, [phenol] =25 mg/L	240	86.0%	0.381	Removal 90% after 3 cycles	[14]
RGO	[catalyst] = 0.5 g/L, [PS] = 0.8 mM, [phenol] =25 mg/L	240	86.0%	0.317	Removal 90% after 3 cycles	[14]
NCd-4-800	[catalyst] = 0.5 g/L, [PS] = 0.5 mM, [phenol] =10 mg/L	30	85%	— [a]	Removal 62% after 3 cycles	[15]
N-BC900	[catalyst] = 0.2 g/L, [PS] = 2 mM, [phenol] =20 mg/L	20	100%	0.206	Removal 45% after 5 cycles	[16]
Cu-GBC10C	[catalyst] = 0.3 g/L, [PS] = 2 mM, [phenol] =0.1 mM	30	90%	— [a]	Removal 30.6% after 4 cycles	[17]
BC-24	[catalyst] = 0.1 g/L, [PS] = 0.5 mM, [phenol] =0.2 mM	25	100%	0.683	Removal 30% after 3 cycles	This work

[a] Not given.

References

1. Qiu, Z.; Wang, Y.; Bi, X.; Zhou, T.; Zhou, J.; Zhao, J.; Miao, Z.; Yi, W.; Fu, P.; Zhuo, S., Biochar-based carbons with hierarchical micro-meso-macro porosity for high rate and long cycle life supercapacitors. *J. Power Sources* **2018**, *376*, 82-90.
2. Lin, G.; Wang, Q.; Yang, X.; Cai, Z.; Xiong, Y.; Huang, B., Preparation of phosphorus-doped porous carbon for high performance supercapacitors by one-step carbonization. *RSC Adv.* **2020**, *10*, 17768-17776.
3. Dat, N. T.; Tran, T. T. V.; Van, C. N.; Vo, D.-V. N.; Kongparakul, S.; Zhang, H.; Guan, G.; Samart, C., Carbon sequestration through hydrothermal carbonization of expired fresh milk and its application in supercapacitor. *Biomass Bioenerg.* **2020**, *143*, 105836.
4. Jiang, F.; Zhu, Y.; Liu, Z.; Zhang, X.; Ma, W.; Wu, H.; Huang, X.; Zhang, Q., Novel synthesis route for the preparation of mesoporous nitrogen-doped carbons from forestry wastes for supercapacitors. *Surf. Interfaces* **2021**, *24*, 101132.
5. Tang, D.; Luo, Y.; Lei, W.; Xiang, Q.; Ren, W.; Song, W.; Chen, K.; Sun, J., Hierarchical porous carbon materials derived from waste lentinus edodes by a hybrid hydrothermal and molten salt process for supercapacitor applications. *Appl. Surf. Sci.* **2018**, *462*, 862-871.
6. Sudhan, N.; Subramani, K.; Karnan, M.; Ilayaraja, N.; Sathish, M., Biomass-Derived Activated Porous Carbon from Rice Straw for a High-Energy Symmetric Supercapacitor in Aqueous and Non-aqueous Electrolytes. *Energ. Fuel.* **2017**, *31*, 977-985.
7. Xia, C.; Surendran, S.; Ji, S.; Kim, D.; Chae, Y.; Kim, J.; Je, M.; Han, M.-K.; Choe, W.-S.; Choi, C. H.; Choi, H.; Kim, J. K.; Sim, U., A sulfur self-doped multifunctional biochar catalyst for overall water splitting and a supercapacitor from Camellia japonica flowers. *Carbon Energy* **2022**, *4*, 491-505.
8. Ma, Z.-W.; Liu, H.-Q.; Lü, Q.-F., Porous biochar derived from tea saponin for supercapacitor electrode: Effect of preparation technique. *J. Energy Storage* **2021**, *40*, 102773.
9. Yao, S.; Zhang, Z.; Wang, Y.; Liu, Z.; Li, Z., Simple one-pot strategy for converting biowaste into valuable graphitized hierarchically porous biochar for high-efficiency capacitive storage. *J. Energy Storage* **2021**, *44*, 103259.
10. Sun, C.; Chen, T.; Huang, Q.; Zhan, M.; Li, X.; Yan, J., Activation of persulfate by CO_2-activated biochar for improved phenolic pollutant degradation: Performance and mechanism. *Chem. Eng. J.* **2020**, *380*, 122519.
11. Zhang, H.; Tang, L.; Wang, J.; Yu, J.; Feng, H.; Lu, Y.; Chen, Y.; Liu, Y.; Wang, J.; Xie, Q., Enhanced surface activation process of persulfate by modified bagasse biochar for degradation of phenol in water and soil: Active sites and electron transfer mechanism. *Colloids Surf. Physicochem. Eng. Aspects* **2020**, *599*, 124904.
12. Fan, X.; Yu, Y.; Dong, S.; Liu, Y.; Song, C.; Li, Q.; Wang, X., Heteroatoms-doped biochar derived from deciduous resource as persulfate catalysts for efficient degradation of phenol. *J. Water Process Eng.* **2022**, *48*, 102866.
13. Zhao, L.; Zhang, H.; Zhao, B.; Lyu, H., Activation of peroxydisulfate by ball-milled α-FeOOH/biochar composite for phenol removal: Component contribution and internal mechanisms. *Environ. Pollut.* **2022**, *293*, 118596.
14. Wang, S.; Wang, J., Kinetics of PMS activation by graphene oxide and biochar. *Chemosphere* **2020**, *239*, 124812.
15. Xu, L.; Wu, C.; Liu, P.; Bai, X.; Du, X.; Jin, P.; Yang, L.; Jin, X.; Shi, X.; Wang, Y., Peroxymonosulfate activation by nitrogen-doped biochar from sawdust for the efficient degradation of organic pollutants. *Chem. Eng. J.* **2020**, *387*, 124065.
16. Zhu, S.; Huang, X.; Ma, F.; Wang, L.; Duan, X.; Wang, S., Catalytic Removal of Aqueous Contaminants on N-Doped Graphitic Biochars: Inherent Roles of Adsorption and Nonradical Mechanisms. *Environ. Sci. Technol.* **2018**, *52*, 8649-8658.
17. Wan, Z.; Sun, Y.; Tsang, D. C. W.; Yu, I. K. M.; Fan, J.; Clark, J. H.; Zhou, Y.; Cao, X.; Gao, B.; Ok, Y. S., A sustainable biochar catalyst synergized with copper heteroatoms and CO_2 for singlet oxygenation and electron transfer routes. *Green Chem.* **2019**, *21*, 4800-4814.
